# Large-Scale Simulation of the Phenotypical Variability Induced by Loss-of-Function Long QT Mutations in Human Induced Pluripotent Stem Cell Cardiomyocytes

**DOI:** 10.3390/ijms19113583

**Published:** 2018-11-13

**Authors:** Michelangelo Paci, Simona Casini, Milena Bellin, Jari Hyttinen, Stefano Severi

**Affiliations:** 1 Faculty of Biomedical Sciences and Engineering, BioMediTech Institute, Tampere University of Technology, FI-33720 Tampere, Finland; jari.hyttinen@tut.fi; 2 Department of Anatomy and Embryology, Leiden University Medical Center, 2333 ZA Leiden, The Netherlands; s.casini@amc.uva.nl (S.C.); m.bellin@lumc.nl (M.B.); 3 Department of Electrical, Electronic and Information Engineering “Guglielmo Marconi”, University of Bologna, 40126 Cesena, Italy; stefano.severi@unibo.it

**Keywords:** action potential, disease model, induced pluripotent stem cell-derived cardiomyocyte, in silico drug tests, in silico model, long QT, *population of models*

## Abstract

Loss-of-function long QT (LQT) mutations inducing LQT1 and LQT2 syndromes have been successfully translated to human induced pluripotent stem cell-derived cardiomyocytes (hiPSC-CMs) used as disease-specific models. However, their in vitro investigation mainly relies on experiments using small numbers of cells. This is especially critical when working with cells as heterogeneous as hiPSC-CMs. We aim (i) to investigate in silico the ionic mechanisms underlying LQT1 and LQT2 hiPSC-CM phenotypic variability, and (ii) to enable massive in silico drug tests on mutant hiPSC-CMs. We combined (i) data of control and mutant slow and rapid delayed rectifying K^+^ currents, I_Kr_ and I_Ks_ respectively, (ii) a recent in silico hiPSC-CM model, and (iii) the *population of models* paradigm to generate control and mutant populations for LQT1 and LQT2 cardiomyocytes. Our four populations contain from 1008 to 3584 models. In line with the experimental in vitro data, mutant in silico hiPSC-CMs showed prolonged action potential (AP) duration (LQT1: +14%, LQT2: +39%) and large electrophysiological variability. Finally, the mutant populations were split into normal-like hiPSC-CMs (with action potential duration similar to control) and at risk hiPSC-CMs (with clearly prolonged action potential duration). At risk mutant hiPSC-CMs carried higher expression of L-type Ca^2+^, lower expression of I_Kr_ and increased sensitivity to quinidine as compared to mutant normal-like hiPSC-CMs, resulting in AP abnormalities. In conclusion, we were able to reproduce the two most common LQT syndromes with large-scale simulations, which enable investigating biophysical mechanisms difficult to assess in vitro, e.g., how variations of ion current expressions in a physiological range can impact on AP properties of mutant hiPSC-CMs.

## 1. Introduction

The development of induced pluripotent stem cell (iPSC) technology provides the opportunity for generating and culturing iPSC-derived cardiomyocytes obtained from human material (hiPSC-CMs). An important application of hiPSC-CMs is the assessment of drug cardiotoxicity [1]. In recent years, their use in the field of safety pharmacology has been supported by comprehensive in vitro proarrhythmia assay (CiPA), which aims to combine the in vitro drug assays on hiPSC-CMs with in silico simulations [2]. Furthermore, hiPSC-CMs have been able to recapitulate the phenotype of several inherited cardiac arrhythmia syndromes such as LQT1 [3], LQT2 [4,5], LQT3 [6], LQT14 [7], catecholaminergic polymorphic ventricular tachycardia [8], hypertrophic cardiomyopathy [9] and Brugada Syndrome [10].

In 2013, we published the first in silico hiPSC-CMs model [11], based on our previous model of cardiomyocyte derived from human embryonic stem cells [12] and on the experimental data by Ma et al. [13]. As recently acknowledged by the FDA [14] and the pharmaceutical industry, in vitro tests on cardiac cells, in particular hiPSC-CMs, certainly benefits from the use of such in silico models of the cardiac cellular electrophysiology [15]. This is true for the screening of new compounds to be tested for cardiac safety, as well as for the study of the ion channel biophysical properties underlying the electrophysiology of control and diseased cells. The main advantage of integrating reliable in silico models in the standard in vitro laboratory practice is the possibility of running huge amount of simulations in shorter time than in vitro experiments. Moreover, in silico models are white boxes, therefore all the internal mechanisms representing the cellular biophysics can be observed without altering the system behavior and be tuned or modified in an easier way than a real biological system. Specifically for hiPSC-CMs, the use of advanced in silico modeling techniques are extremely important to manage one of the main hiPSC-CM challenges, i.e., their electrophysiological variability observed in vitro [16]. The *population of* in silico *models* [17,18] approach enables the development of a huge ensemble of in silico models which, after experimental calibration, mimics the behavior of the in vitro population of cells, with all the aforementioned advantages of model usage. In this case, the added value consists of having an in silico cell population that is way larger than whatever population could be obtained or analyzed in vitro. As a matter of fact, the literature presents electrophysiological investigations on hiPSC-CMs performed on samples containing only a few cells, while an in silico population contains at least hundreds of models. We recently used this modeling technique to investigate the LQT3 syndrome effect on in silico hiPSC-CMs and to predict the effects of two drugs, commonly used in this syndrome treatment, also elucidating possible mechanisms for the development of adverse drug effects [18]. The same approach was used also by Passini et al. [19] for a comprehensive in silico drug trial on 62 compounds, on in silico populations of adult ventricular cardiomyocytes, which obtained higher accuracy than studies involving animal models.

The aim of this work consist of exploiting a recently published model of the electrophysiology of hiPSC-CMs (Paci2018) [20] and the *population of* in silico *models* approach to replicate the behavior of the two most common LQT forms: LQT1 and LQT2. Both forms of LQT can result in a dangerous prolongation of the duration of the cardiomyocyte action potential (AP), which makes the cells prone to develop arrhythmias. At the whole organ level, this reflects in a prolongation of the QT segment in the electrocardiogram, which is associated with dramatic outcomes, such as syncope, and sudden cardiac death due to ventricular tachyarrhythmias [21]. LQT1 is associated with loss-of-function mutations in the *KCNQ1* gene, which encodes for the α-subunit of the channel conducting the slowly activating delayed rectifier potassium current (I_Ks_), while LQT2 arises from loss-of-function mutations in *KCNH2* (also known as *hERG*), encoding the rapidly activating delayed rectifier potassium current (I_Kr_). In this work, we focused our in silico analysis on the *KCNQ1* R190Q mutation associated with LQT1 and the *KCNH2* N996I mutation associated with LQT2, both already in vitro characterized in hiPSC-CMs [3,4]. Together with our previous work on LQT3 [18], this work covers the spectrum of the most common forms of LQT syndromes and their in silico simulations based on populations of hiPSC-CMs, remarking the importance of the synergy of in vitro and in silico experiments for disease modeling using hiPSCs.

## 2. Results

In this work, we produced four populations of in silico hiPSC-CM models showing spontaneous electrical activity, as detailed in Section 4.4 and Section 4.5 of Materials and Methods. In particular, the LQT1_CTRL and LQT1_MUT populations were based on the Paci2018 model [20], where the original I_Ks_ was replaced with the control and mutant I_Ks_ from Moretti et al. [3]. Likewise, in the LQT2_CTRL and LQT2_MUT populations, I_Kr_ was replaced with the control and mutant I_Kr_ described in Bellin et al. [4]. To facilitate the comparison with the in vitro experiments, LQT2_CTRL and LQT2_MUT were also paced at 60 bpm. To generate the LQT1_CTRL and the LQT2_CTRL populations, we sampled thirteen parameters in the baseline model. The sampled parameters are the maximum conductances/currents of fast and late Na^+^ currents (I_Na_, I_NaL_), funny current (I_f_), L-type Ca^2+^ current (I_CaL_), I_Ks_, I_Kr_, inward rectifying K^+^ current (I_K1_), transient outward K^+^ current (I_to_), Na^+^/Ca^2+^ exchanger (I_NCX_), Na^+^/K^+^ pump (I_NaK_), sarcolemmal Ca^2+^ pump (I_pCa_), SERCA pump (I_SERCA_) and RyR-sensitive Ca^2+^ release from sarcoplasmic reticulum (I_RyR_). Each parameter was sampled in the range [0.5; 2] of its nominal value from [20], thus obtaining two random populations. Each random population was then calibrated using AP biomarker values experimentally recorded on spontaneous hiPSC-CM APs, thus obtaining the populations LQT1_CTRL and LQT2_CTRL. A more detailed description of this method is reported in Section 4 and a summary of this approach is reported in Figure S1. The number of models included in each population is reported in Table 1.

### 2.1. The Control and the Mutant LQT1 Populations

**Figure 1:**
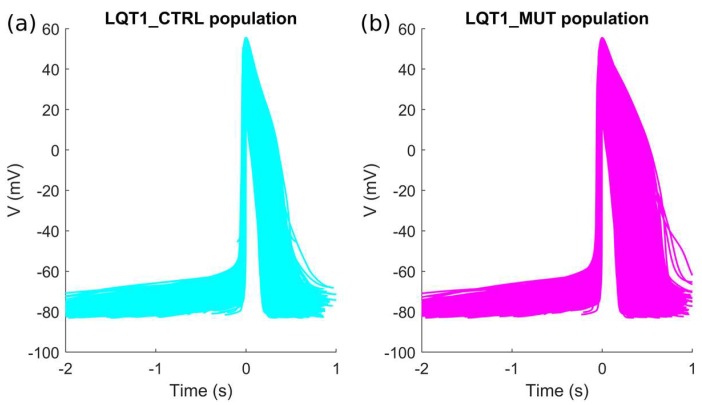
Action potentials included in the two populations built using the I_Ks_ experiments by Moretti et al. [3]: (**a**) LQT1_CTRL; (**b**) LQT1_MUT. A magnified version of this figure is reported as Figure S2 in the Supplementary Material.

**Figure 2:**
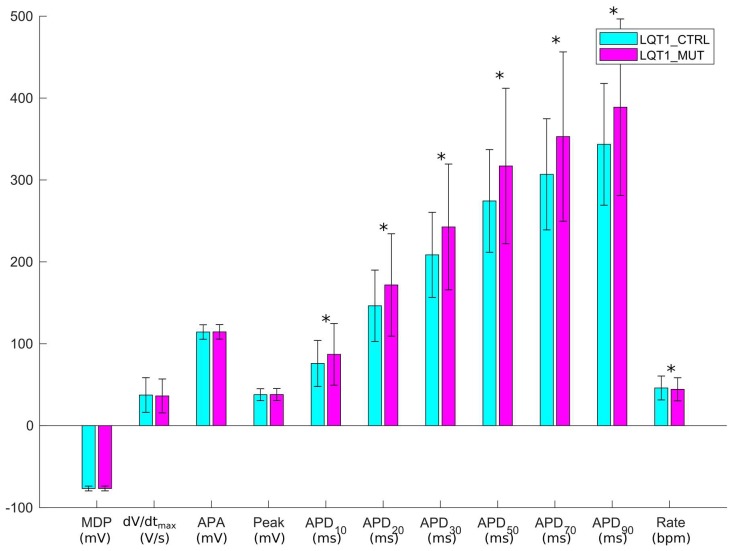
Comparison of the AP mean biomarkers of LQT1_CTRL (cyan) and LQT1_MUT (magenta). Error bars represent the standard deviation. Significant differences in the AP biomarkers are marked with a black star * (Mann-Whitney *U*-test, *p*-value < 0.01).

**Figure 3:**
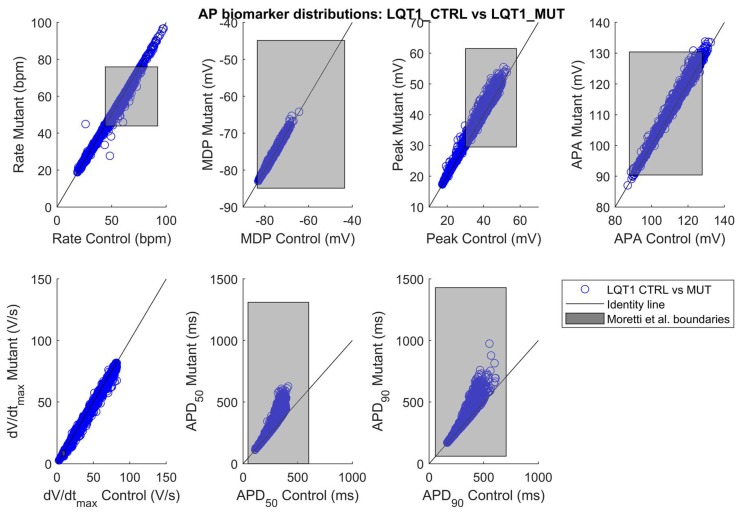
AP biomarker distributions in the populations LQT1_CTRL vs. LQT1_MUT (blue circles). The black lines represent the identity lines, i.e., when the AP biomarkers are equals in LQT1_CTRL and LQT1_MUT. The grey rectangles represents the boundaries built on the experimental data from the Moretti 2010 [3] control and mutant datasets, obtained in spontaneously beating hiPSC-CMs (see Table 2): lower bound (mean − 2SD) and upper bound (mean + 2SD) for each AP biomarker.

**Figure 4:**
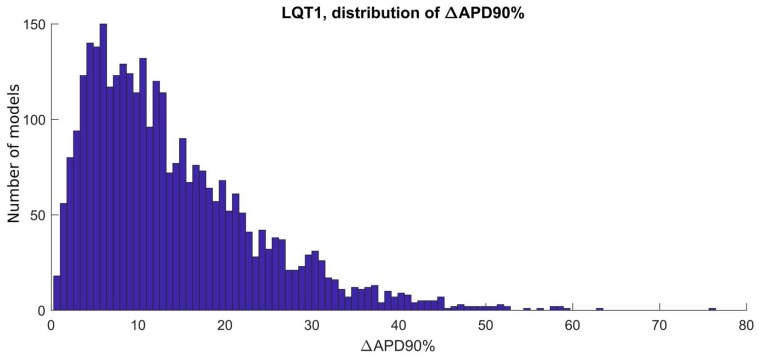
Distribution of the APD_90_ changes due to LQT1 in spontaneous APs, expressed as percent of the APD_90_ control value. The average ΔAPD_90_% is +14%.

Out of the initial 10,000 parameter sets (i.e., 10,000 putative/candidate cell models) obtained by Latin Hypercube sampling, only 3584 produced models which satisfied the experimental calibration represented by the filtering conditions listed in Section 4.4. APs from the 3584 hiPSC-CM models included in the LQT1_CTRL population are shown in Figure 1a and Figure S2a. After “switching on” the mutation in the 3584 models, we obtained the LQT1_MUT population, which contains 3238 models. 346 models were not included in the LQT1_MUT population for one or more of the reasons listed in Section 4.5. The APs produced by the LQT1_MUT population are shown in Figure 1b and Figure S2b, from which an overall AP prolongation can be appreciated. Figure 2 compares the AP biomarkers computed on the spontaneous APs included in LQT1_CTRL (cyan) and LQT1_MUT (magenta). Clearly, the mutant population shows considerably prolonged APs. In Figure 3, we report the scatter plots of the AP biomarkers in LQT1_CTRL vs. the AP biomarkers in LQT1_MUT. The grey rectangles were built using the AP biomarkers reported in Table 2, to show how our populations cover the biomarker space from the Moretti 2010 dataset (see Table 2) [3]. For each AP biomarker, the lower bound is computed as mean—2SD and the upper bound as mean + 2SD. Of note, such boundaries were not used to calibrate the LQT1_MUT population (since we turned on the mutation on all the 3584 models of LQT1_CTRL), but they are here reported for comparison. The AP biomarkers which are mainly affected by the LQT1 mutation are the AP duration at 50% of repolarization (APD_50_) and the AP duration at 90% of repolarization (APD_90_). In particular, the longer is the AP in control conditions, the longer is the AP prolongation induced by the mutation, as shown by the clouds of APD_50_ and APD_90_ points that detach from the identity lines for long control APD values. An alternative representation of the AP biomarker distributions in LQT1_CTRL and LQT1_MUT is reported in Figure S3. Finally, Figure 4 reports the distribution of the APD_90_ changes expressed as percent of the control APD_90_ (ΔAPD_90_%). The average simulated ΔAPD_90_% is +14%, while the experimental one is +95%. To compare LQT1_MUT with LQT1_CTRL, we included in this analysis only the control and mutant versions of the 3238 models included in LQT1_MUT.

### 2.2. The Control and the Mutant LQT2 Populations

**Figure 5:**
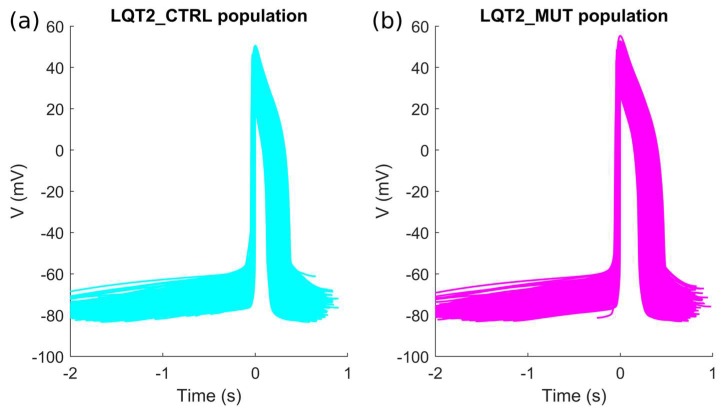
Action potentials included in the two populations built using the I_Kr_ experiments by Bellin et al. [4]: (**a**) LQT2_CTRL; (**b**) LQT2_MUT. A magnified version of this figure is reported as Figure S4 in the Supplementary Material.

**Figure 6:**
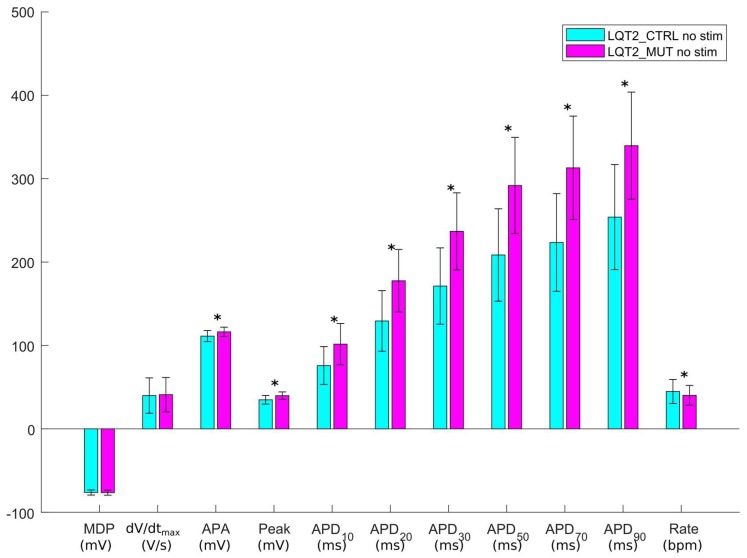
Comparison of the AP mean biomarkers computed on spontaneous APs in LQT2_CTRL (cyan) and LQT2_MUT (magenta). Error bars represent the standard deviation. Significant differences in the AP biomarkers are marked with a black star * (Mann-Whitney *U*-test, *p*-value < 0.01).

**Figure 7:**
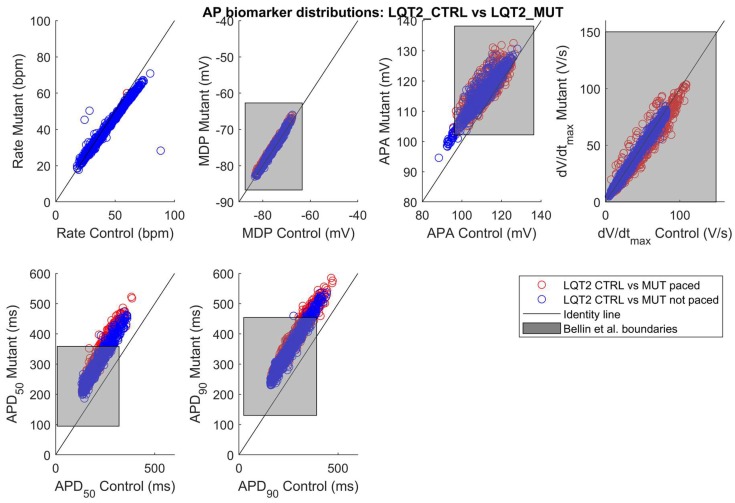
AP biomarker distributions in the populations LQT2_CTRL vs. LQT2_MUT in the non-paced (blue circles) and paced (red circles) cases. The black lines represent the identity lines, i.e., when the AP biomarkers are equals in LQT2_CTRL and LQT2_MUT. The grey rectangles represents the boundaries built on the experimental AP biomarkers from the Bellin 2013 control and mutant datasets [4] recorded in paced hiPSC-CMs and reported in Table 3: lower bound (mean − 2SD) and upper bound (mean + 2SD) for each AP biomarker except for the Rate biomarker (pacing rate set to 60 bpm).

**Figure 8:**
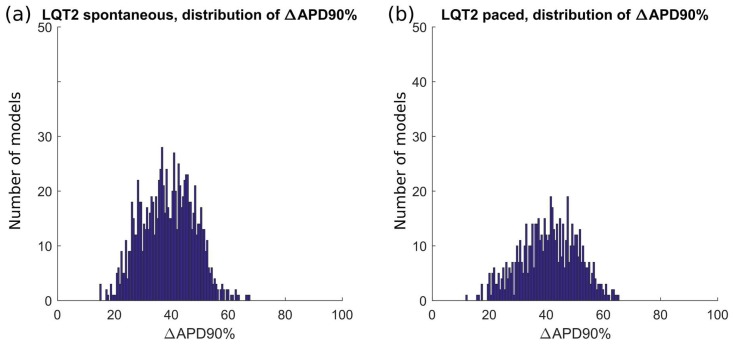
Distribution of the APD_90_ changes due to the LQT2 mutation, expressed as percent of the APD_90_ control value. The average ΔAPD_90_% is +39% and +41% for the in silico spontaneous (**a**) and externally paced (**b**) hiPSC-CMs, respectively.

As in the previous section, only a limited number of parameter sets out of the initial 10,000 were included in the LQT2_CTRL population: 1226. In Figure 5a and Figure S4a, we show the spontaneous APs of the LQT2_CTRL population. After switching on the LQT2 mutation in these 1226 models, a subset of 1008 models was included in the LQT2_MUT population, shown in Figure 5b and Figure S4b. The comparison of the AP biomarkers is reported in Figure 6, showing also in this case a substantial prolongation of the APD in the mutant population. Since the data reported in Bellin et al. [4] (see Table 3) refer to APs measured in hiPSC-CMs stimulated at 60 bpm, we also stimulated LQT2_CTRL and LQT2_MUT, obtaining 979 and 650 models respectively. This discrepancy between the spontaneous and stimulated LQT2_CTRL and LQT2_MUT populations is due to the fact that subsets of models (247 and 358 models, respectively) were not able to synchronize their spontaneous APs with the external pacing. This lack of synchronization led to scenarios where a spontaneous AP was triggered before the external stimulus, which eventually occurred in the middle of the spontaneous AP, consequently making computing the AP biomarkers meaningless. Figure 7 reports the scatter plots of the AP biomarkers in LQT2_CTRL vs. the AP biomarkers in LQT2_MUT, in non-paced (blue circles) and paced (red circles) conditions. The grey rectangles represent the experimental AP biomarkers boundaries built on the data from paced hiPSC-CMs reported in Table 3, as for Figure 3 in the previous Section 2.1. The AP biomarkers affected the most by the LQT2 mutation are APD_50_ and APD_90_, which are prolonged by almost the same extent, independently of the APD in control conditions. An alternative representation of the AP biomarker distributions in LQT2_CTRL and LQT2_MUT is reported in Figure S5. Figure 8 shows the distribution of ΔAPD_90_% in case of spontaneous APs (Figure 8a) or paced APs (Figure 8b). The average simulated ΔAPD_90_% is +39% when computed on the spontaneous APs, while it is +41% when computed on the paced APs, respectively. The experimental ΔAPD_90_% reported by Bellin et al. [4] is +41% in conditions of external pacing.

### 2.3. At Risk vs. Normal-Like Mutant hiPSC-CMs

**Figure 9:**
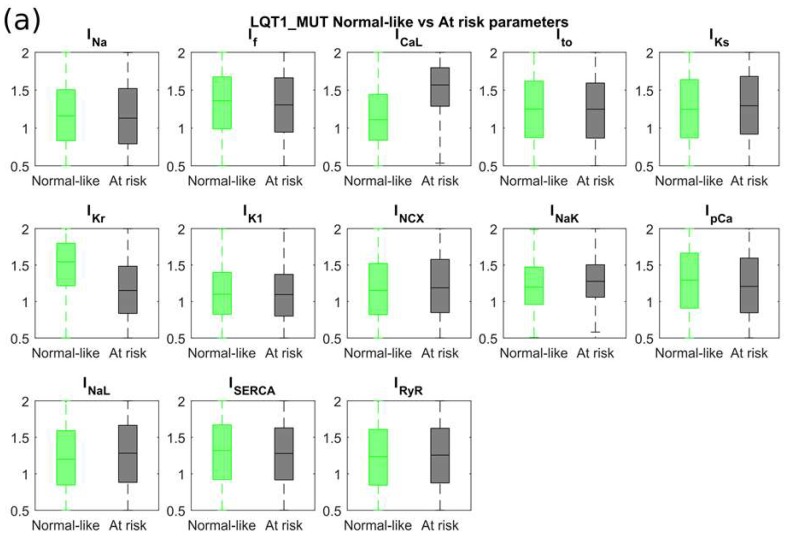

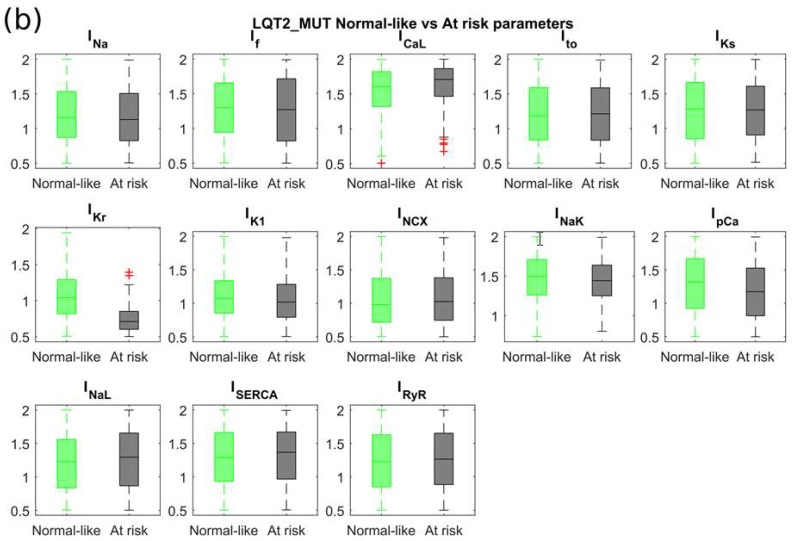
Distribution of the sampling coefficients of the maximum conductances/currents (see Section 4.4) for the main ionic currents in the at risk and normal-like groups into which LQT1_MUT and LQT2_MUT were split. For each population, we considered a median difference of at least 10% to identify the main ionic mechanisms that differ between the two groups. (**a**) LQT1_MUT: at risk models exhibited smaller I_Kr_ (−25%) and larger I_CaL_ (+41%). (**b**) LQT2_MUT: at risk models exhibited smaller I_Kr_ (−32%) and I_pCa_ (−11%). Outliers are represented as red crosses.

The mutant populations LQT1_MUT and LQT2_MUT were split into two groups by means of k-means clustering, exploiting APDs and Rate biomarkers, as detailed in Section 4.5. Provided that the *KCNQ1* R190Q and the *KCNH2* N996I mutations affect all cells in LQT1_MUT and LQT2_MUT, respectively, we named as normal-like those models with APDs similar to control hiPSC-CMs, and at risk those models with prolonged APDs. Figure 9 shows the different distributions of the maximum conductances/currents for the normal-like and at risk models of populations LQT1_MUT (Figure 9a) and LQT2_MUT (Figure 9b), respectively. For each of these mutant populations, we considered a median difference of at least 10% to identify the main ionic mechanisms that differ between the at risk and normal-like groups. At risk models in LQT1_MUT exhibited (i) smaller I_Kr_ (outward current, −25%) and (ii) larger I_CaL_ (inward current, +41%). In LQT2_MUT, again the at risk group showed reduced I_Kr_ (−32%) with the addition of a reduced I_pCa_ (outward current, −11%). Notably, in contrast to the LQT1_MUT at risk models, at risk models in LQT2_MUT showed only a moderately larger I_CaL_ (+6%). These differences in ionic current expressions are emblematic of a reduced repolarization reserve in the at risk groups. With the term repolarization reserve, we refer “*to the idea is that the complexity of repolarization includes some redundancy. As a consequence, loss of one component (such as I_Kr_ or I_Ks_) ordinarily will not lead to failure of repolarization (i.e., marked QT prolongation); as a corollary, individuals with subclinical lesions in other components of the system, say I_Ks_ or calcium current, may display no QT change until I_Kr_ block is superimposed*” (definition adapted from [22,23]).

**Figure 10:**
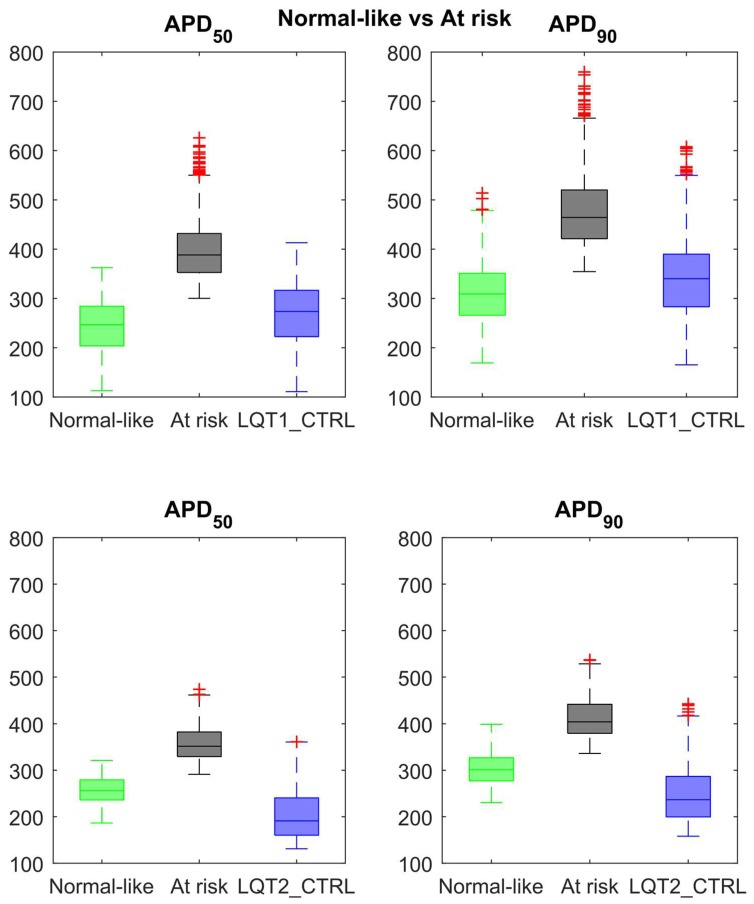
Distribution of APD_50_ (green) and APD_90_ (black) among the at risk and normal-like groups for the LQT1_MUT (**upper row**) and LQT2_MUT (**lower row**). The AP biomarker distribution in the corresponding control population is reported in blue. Outliers are represented as red crosses.

We also run an additional Mann-Whitney *U*-test to identify if other ionic currents could have shown significant differences (*p*-value < 0.01) among the at risk and normal-like groups. No additional differences in currents were identified in the LQT2 at risk group as compared to the normal-like. Conversely, in LQT1_MUT, the at risk group showed significantly larger I_NCX_, I_NaK_ and I_NaL_ and smaller I_pCa_ (median difference compared to normal-like +3%, +7%, +7% and −7% respectively). Figure 10 shows how APD_50_ and APD_90_ of the normal-like groups are way closer to control values than to the computed APD_50_ and APD_90_ of the at risk groups. Furthermore, we evaluated in the control populations LQT1_CTRL and LQT2_CTRL whether the control models corresponding to at risk vs. normal-like mutant models displayed differences in AP biomarker (quantified as median values). Control models leading to at risk LQT1 models displayed increased Peak voltage (+18%) and prolonged APD_50_ and APD_90_ (+42% and +36%, respectively) compared to those leading to normal-like LQT1 models after switching on the I_Ks_ mutation. Similarly, control models leading to at risk LQT2 models were characterized by decreased dV/dt_max_ (–21%), increased Peak voltage (+14%) and prolonged APD_50_ and APD_90_ (+55% and +47%, respectively) compared to control models leading to normal-like LQT2 models after switching on the I_Kr_ mutation.

### 2.4. Quinidine Effect on hiPSC-CMs

**Figure 11:**
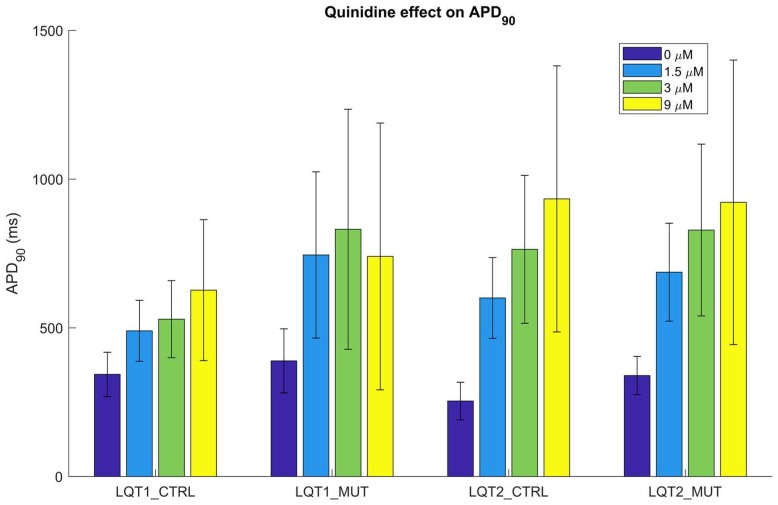
APD_90_ quinidine–induced prolongation in the four in silico populations. APD_90_ values were computed only for the models that did not develop abnormalities in response to quinidine administration. Error bars represent the standard deviation.

**Figure 12:**
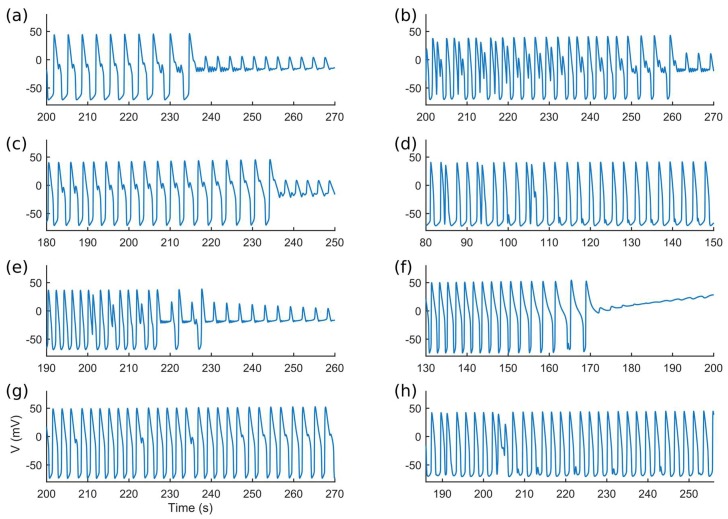
Abnormalities induced by quinidine. (**a**) Single and multiple EADs and repolarization failure. (**b**) Single and multiple EADs and repolarization failure. (**c**) Single EADs and repolarization failure. (**d**) Anticipated APs, EADs and DAD-like abnormalities. (**e**) Multiple EADs and repolarization failure. (**f**) Repolarization failure. (**g**) Single EADs. (**h**) Anticipated APs, EADs and DAD-like abnormalities.

Quinidine, a pro-arrhythmic drug causing AP prolongation, induced a remarkable prolongation of APD_90_ in all four in silico populations. Figure 11 shows quinidine effect on APD_90_ for those models for which computing the AP biomarkers was still possible after drug administration. Moreover, quinidine administration triggered many arrhythmic events, especially at the highest dose (Table 4). Among the models developing abnormalities, we observed many different arrhythmogenic patterns, including early afterdepolarization (EADs), repolarization failure and delayed afterdepolarization (DADs)-like abnormalities (which could also degenerate in anticipated beats), as shown in Figure 12. Finally, Table 4 also reports the percent of at risk and normal-like models in the populations LQT1_MUT and LQT2_MUT that produced abnormalities after quinidine administration (in brackets), showing that at risk models are more prone to abnormalities than the normal-like ones, except for the highest dose, when the amount of abnormalities becomes comparable.

## 3. Discussion

The potential of hiPSC-CMs as disease-specific in vitro models has been clear for a decade. In this work, we showed a synergistic approach between experimental data from cells with two specific ion channel mutations (namely the *KCNQ1* R190Q mutation associated with LQT1 and the *KCNH2* N996I associated with LQT2) and a recent in silico modeling technique, i.e., the *populations of models*. Starting from our recent Paci2018 hiPSC-CM in silico model [20], we were able to integrate patch clamp data of control and mutant I_Ks_ [3] and control and mutant I_Kr_ [4], to produce four in silico populations of control and mutant hiPSC-CMs (LQT1_CTRL, LQT1_MUT, LQT2_CTRL and LQT2_MUT). The hiPSC-CM models included in the aforementioned populations represents single isolated cells; therefore, we did not consider any coupling (e.g., via gap junctions) between the cells of each population.

**Figure 13:**
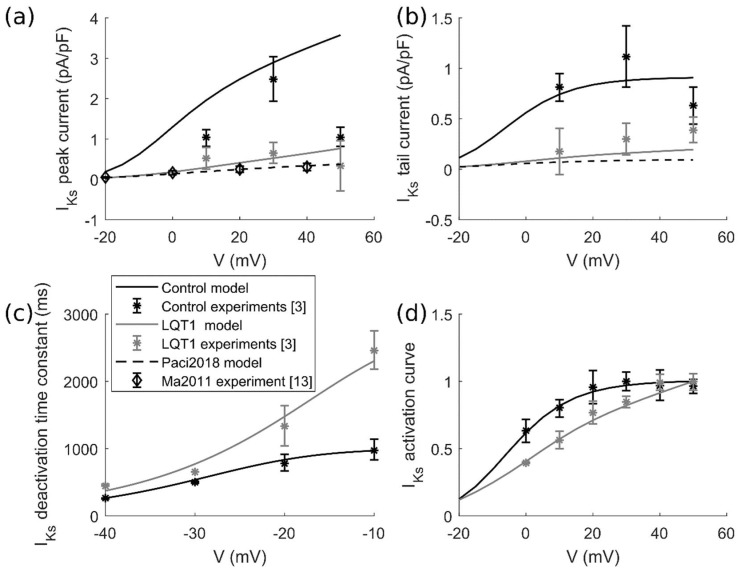
Fitting of the I_Ks_ experimental data from Moretti et al. [3]. The black stars and curve represent the Moretti et al. control data [3] and fitting, respectively. The grey stars and curve represent the Moretti et al. mutant data [3] and fitting, respectively. The diamonds and the dashed curve represent the I_Ks_ measurements from Ma et al. [13] and the I_Ks_ simulated by the Paci2018 model [20]. (**a**) I_Ks_ peak current (a zoom on the Paci2018 I_Ks_ peak current is reported in Figure S7). (**b**) I_Ks_ tail current. (**c**) Voltage dependence of I_Ks_ deactivation kinetics. (**d**) Voltage dependence of I_Ks_ activation.

Firstly, we were able to replicate in silico the main AP feature at cellular level of both the LQT types we investigated, i.e., the prolongation of the AP duration (Figure 2 and Figure 6). In [3], a very large APD_90_ prolongation (+95%) due to LQT1 was observed. In our populations, we did not observe such extreme prolongation, with only few models prolonging APD_90_ over 50% (Figure 4). We ascribe such differences in the effect of the mutation to a quite small sensitivity of the original Paci2018 model [20] to I_Ks_, which reflected the low sensitivity of hiPSC-CMs to I_Ks_ block experimentally recorded by Ma et al. [13], which is actually similar to those of adult myocytes. In fact, in [20], we showed only very small changes in APD_90_ by blocking I_Ks_ up to 90% (ΔAPD_90_ = 18 ms when paced at 1 Hz and ΔAPD_90_ = 13 ms considering spontaneous APs). The replacement of the Moretti et al. control I_Ks_ [3] in the Paci2018 model [20], which is actually greater than the original I_Ks_, as shown in Figure 13a, was indeed sufficient to make the model more sensitive to I_Ks_ block (ΔAPD_90_ = 42 ms, +12%, when blocking 90% I_Ks_) and to I_Ks_ loss-of-function.

The I_Ks_ contribution to repolarization of cardiac membrane potential is highly debated. In Lemoine et al. [24], it was recently shown that a strong I_Ks_ block obtained by 1 µM HMR-1556 did not induce prolongation nor in human left ventricle tissue nor in engineered heart tissue (EHT) samples obtained from hiPSC-CMs, unless the samples were pretreated with I_Kr_ blocker E4031 and in conditions of β-adrenergic stimulation. Even though the results of Lemoine et al. [24] focus on I_Ks_ block and not I_Ks_ loss-of-function due to specific mutations, they seem in contrast with the huge APD prolongation observed by Moretti et al. [3] as consequence of the *KCNQ1* R190Q mutation. A more moderate APD_90_ prolongation (about +28%), associated with more positive APA and elevated plateau phase, was observed by Zhang et al. [25] in hiPSC-CMs carrying the *KCNQ1* c.1781G>A mutation. However, Christ et al. [26] criticized these results and the idea that “*repolarization reserve in hiPSC-CM is profoundly different from native human ventricular cardiomyocytes, with a much larger impact of unstimulated I_Ks_ compared with other potassium currents*” (Quoted from [26]). Conversely, Christ et al. [26] pointed out that the higher APA and elevated plateau phase reported by Zhang et al. [25] could only be explained by the suppression of a very rapidly activated repolarizing current, which is in contrast with the slow I_Ks_ activation kinetics observed in native cardiomyocytes. The different APD prolongations reported upon reduction of I_Ks_ could be ascribed to different maturity degrees of the analyzed hiPSC-CMs. Indeed, hiPSC-CMs in the EHT format are rather mature compared with the two-dimensional differentiation from which single cells were measured in Moretti et al. [3] and in Zhang et al. [25]. It is likely that in immature cells, I_Ks_ plays a major role in AP repolarization, even without adrenergic stimulation, while in native cardiomyocytes as well in more mature hiPSC-CMs (where not only all the other cardiac ion currents are more similar to native myocytes, but also the myocyte structure and the calcium handling properties are improved), the role of I_Ks_ is minimal under baseline conditions. Moreover, it should also be considered that in in vitro studies, the choice of control influences very much the changes that can be measured. In fact, in Moretti et al. [3], an unrelated control hiPSC line was used as reference. However, when the LQT1 mutation was corrected and an isogenic hiPSC control line created [27], a smaller APD prolongation was measured in the LQT1 mutant cardiomyocytes compared to the LQT1 corrected cardiomyocytes (+30%). It is worth noting that the approach we used in the present study to simulate the effect of mutation (hence only by substitution of the I_Ks_ formulation) is definitely more similar to the isogenic situation than to that reported in the original Moretti et al. [3] study. Our in silico analysis predicts a more moderate LQT1 effect than Moretti et al. [3], Zhang et al. [25] and Chen et al. [27], i.e., an average APD_90_ prolongation equal to +14%. However, a subset of cells in our LQT1_MUT population showed strong APD_90_ prolongation. In Figure S6 we reported the parameter distribution of the LQT1_MUT models with APD_90_ prolongation over 50%. It is clear that these models are characterized by (i) overexpression of inward currents (I_Na_, I_NaL_ and I_CaL_), (ii) strong underexpression of I_Kr_, and (iii) strong overexpression of I_Ks_. Our in silico predictions considered such a configuration of ionic current expressions compatible with higher sensitivity to I_Ks_ loss-of-function/I_Ks_ block in hiPSC-CMs. It is an open question whether this higher sensitivity also corresponds to a lower degree of maturity in hiPSC-CMs. Of course such results would need a solid experimental confirmation. In fact, due to the complexity of this dispute, we can only hope for more detailed characterization of I_Ks_ (but also of the other ionic currents) on control and mutant hiPSC-CMs, taking into account the high hiPSC-CM electrophysiological variability, and therefore the performing of measurements on a high number of cells obtained from different commercial lines and different patients.

**Figure 14:**
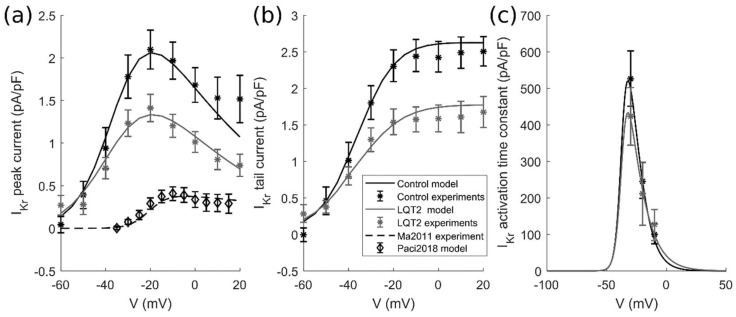
Fitting of the I_Kr_ experimental data from Bellin et al. [4]. The black stars and curve represent the Bellin et al. control data [4] and fitting, respectively. The grey stars and curve represent the Bellin et al. mutant data [4] and fitting, respectively. The diamonds and the dashed curve represent the I_Kr_ measurements from Ma et al. [13] and the I_Kr_ simulated by the Paci2018 model [20]. (**a**) I_Kr_ step current. (**b**) I_Kr_ tail current. (**c**) I_Kr_ activation time constant.

There is more consensus about the role of I_Kr_ in the AP repolarization and the effects of I_Kr_ loss-of-function. Bellin et al. [4] reports an APD_90_ prolongation equal to +41% due to LQT2 in paced hiPSC-CMs [4]. We observed an APD_90_ increment equal to +39% and +41% in LQT2_MUT as compared to LQT2_CTRL, respectively, for spontaneous and paced APs, in full agreement with the experiments. In a previous article, we showed the strong effect that I_Kr_ block has on the original Paci2018 model [20]. The direct replacement of the Bellin et al. control I_Kr_ [4] (clearly larger than the original I_Kr_ reported in dashed in Figure 14) in the original Paci2018 model [20] dramatically shortened APD_90_ (~140 ms) and created an AP shape that was not included in the LQT2_CTRL population and, in cascade, in the LQT2_MUT population. Here, the power of running simulations with populations of in silico models is already clear. In the LQT1 example, starting from a baseline model showing only +11% APD_90_ prolongation, we obtained a population including models with prolongation over 50%. Conversely, in the LQT2 case, starting from a baseline model which was not even included in the control population, we obtained more than 1000 models whose mean ΔAPD_90_ was in agreement with the in vitro experiments [4].

Figure 3, Figure 7, Figure S3 and Figure S5 show how well our populations were able to cover the control and mutant AP biomarkers from the Moretti2010 [3] and Bellin2013 [4] datasets. Notably, from the Moretti2010 dataset, only control AP biomarkers (and not mutant ones) were used for the calibration of the control populations (together with the other five datasets listed in Section 4.4, Table 5). In fact, our approach consisted of (i) calibrating the two control populations using AP biomarker ranges from the six experimental datasets of AP biomarkers computed on spontaneous hiPSC-CM APs joined together, and (ii) switching on the mutations in all the models we included in the control populations. This approach, already used in [18,28], has the advantage of producing more control phenotypes, compared to calibrating the control population only with a single dataset, e.g., Moretti2010 [3]. Secondly, switching on the mutations only in the models included in the control populations, allows a direct comparison between each mutant and control cell, e.g., to assess APD_90_ prolongation for each cell. Notably, we could not use the data from Bellin et al. [4] for calibration, since they were recorded in paced hiPSC-CMs, while we calibrated our control populations on spontaneous AP data. The APD_90_ scatter plots in Figure 3 and Figure 7 highlight that the two mutations affect APD with completely different patterns. While in LQT2_MUT APD_90_ is always prolonged by almost the same extent (Figure 7), independently of the APD in control conditions, in LQT1_MUT there is a marked sensitivity and the longer APD is in control, the larger its prolongation due to the mutation will be (Figure 3). This is not surprising; given the slow activation kinetic of I_Ks_, its contribution (and the impact of its reduction) to repolarization is expected to increase with APD. This is less true for I_Kr_, whose action is mainly mediated by rapid recovering from inactivation during phase 3 of the AP. Moreover, from the last scatter plot in Figure 3, it is evident that, for long APD, LQT1_MUT is characterized by very large intersubject variability, with very different mutant APD_90_ values in correspondence of the same control APD_90_. This points to the fact that in LQT1_MUT (much more than LQT2_MUT) the effect of the mutation could be compensated or exacerbated by over/under-expression of the other ionic currents.

We used a technique previously described [18] to discriminate between at risk and normal-like mutant hiPSC-CMs by means of an unsupervised classifier. Figure 10 shows how the normal-like group in both LQT1_MUT and LQT2_MUT exhibits AP duration closer to that observed in control, despite the mutation. We identified a subset of ionic currents whose overexpression or underexpression could act as protective mechanisms against the LQT mutations investigated in this paper. In both LQT1_MUT and LQT2_MUT, the main protective mechanisms were the overexpression of I_Kr_ and underexpression of I_CaL_ (to a lesser extent in LQT2_MUT) and I_pCa_. It is interesting to note that in LQT1_MUT, the at risk group showed also significant overexpression of I_NCX_ and I_NaK_, as compared to LQT1_CTRL. This is actually in agreement with the work of Krogh-Madsen et al. [29], where the authors used two different genetic optimizations to create two models (Multi-variable and APD_LQT_), able to summarize the three most common forms of LQT, i.e., types 1, 2 and 3. In both the Multi-variable and APD_LQT_ models, the parameter optimization resulted in an overexpression of I_NCX_ (3.05× and 1.75×) and I_NaK_ (1.91× and 7.4×). Therefore, our simulations suggest that underexpression of I_NaK_ and I_NCX_ could act as well as protective mechanisms against the mutation effects.

Finally, we also provided a simple but powerful application of our in silico populations, i.e., an illustrative drug test. We chose quinidine, known to prolong APD [30] and to be a high-risk drug for Torsades de Pointes (a malignant type of arrhythmia, associated with sudden cardiac death) generation [19,31]. Such an aggressive drug is expected to trigger many arrhythmogenic events, both in the mutant and in the control populations. It is interesting to observe that the differences in APD prolongation between LQT2_CTRL and LQT2_MUT, tend to disappear by increasing the quinidine dose (Figure 11). This is due to the fact that LQT2_CTRL and LQT2_MUT differ only for the I_Kr_ formulation, which is the most affected current by quinidine (Table S1). In principle, the full I_Kr_ block should lead to the same APD_90_ in both LQT2_CTRL and LQT2_MUT, as shown in Figure 11 in corrispondence of the higher dose of quinidine. The same does not happen for LQT1_CTRL and LQT1_MUT. In fact, these two populations differ for their I_Ks_ formulation, which is affected by quinidine to a smaller extent (Table S1). Nevertheless, in LQT1_MUT the compromised repolarization reserve amplifies the effect of the I_Kr_ block due to quinidine (Figure 11). This is visible also from the increasing difference in the amount of AP abnormalities occurring in LQT1_MUT as compared to LQT1_CTRL at increasing drug doses. Such differences do not emerge in LQT2_CTRL and LQT2_MUT (Table 4), due to the presence of high I_Kr_ intensity in both populations (Figure 14a). In fact, APs are surely prolonged in LQT2_CTRL and LQT2_MUT as a consequence of drug administration; however, they are less prone to develop abnormalities as compared to LQT1_CTRL and LQT1_MUT.

The onset of afterdepolarizations or beating arrest as a response to high doses of quinidine was experimentally demonstrated by Kuusela et al. [30] on hiPSC-CM aggregates, whose field potentials were recorded by means of multielectrode arrays. Quinidine was administrated to hiPSC-CM clusters derived from two wild-type lines and three LQT lines (namely LQT1a, LQT1b and LQT2). All hiPSC-CM clusters showed afterdepolarizations and beating arrest. When we consider only the models that did not react to quinidine with arrhythmic events, the APD_90_ prolongation was quite extreme for all the four in silico populations: at the highest doses APD_90_ was more than doubled (Figure 11). A more moderate field potential duration prolongation (+14–16%, at 9 µM) was observed in control and mutant clusters in [30]. We ascribe this difference in prolongation to the following factors. Firstly, the number of cell clusters used in Kuusela et al. [30] is limited (min 6, max 16). Secondly, we performed our simulations on single in silico cells and not on cell clusters; therefore, we do not consider any interaction between hiPSC-CMs or between hiPSC-CMs and cells that are not cardiomyocytes but that could still be present in aggregates, potentially affecting the AP depolarization and repolarization phases and mitigating the drug effect [32]. Coupling in multicellular systems (e.g., cardiomyocyte-cardiomyocyte or cardiomyocyte-fibroblast coupling) affects the cell electrophysiology, as we also reported in Paci et al. [12], and some averaging of the population of responses is expected. However, a quantitative evaluation of the effects of coupling is beyond the aim of this study. Finally, quinidine was simulated with the simple single-pore block model, which is effective in showing the main effects of the drug (including the onset of arrhythmic events), but which does not describe more complex effects, e.g., due to state dependent interactions between the drug and the ion channels.

Finally, in this study, we chose to develop two control models to be used as stems to generate the LQT1_CTRL and the LQT2_CTRL populations, instead of a single control model containing both the Moretti et al. [3] control I_Ks_ and the Bellin et al. [4] control I_Kr_. This choice is justified by the fact that both I_Ks_ from Moretti et al. [3] and I_Kr_ from Bellin et al. [4] are extremely stronger than the original I_Ks_ and I_Kr_ in the Paci2018 model [20]. Therefore, a single control model obtained by replacing both the control I_Ks_ and I_Kr_ reported in Figure 13 and Figure 14, would have generated a baseline with an extremely short APD (~105 ms), making it very hard to generate a control population covering both the two experimental datasets, that show dramatically different APD values. This would have been particularly problematic when switching on the LQT1 mutation, since the AP prolongation induced by the I_Ks_ loss-of-function would have been hidden by the very strong Bellin et al. control I_Kr_ [4].

## 4. Materials and Methods

### 4.1. General Approach and Study Design

This study was organized in the following steps. Firstly, we fit the experimental voltage clamp data for the control and the mutant I_Ks_ [3] and I_Kr_ [4]. Secondly, for each of the aforementioned K^+^ currents, we inserted its control formulation into the recently published Paci2018 model of hiPSC-CM [20], thus obtaining two baseline models, the former with an updated control I_Ks_, the latter with an updated control I_Kr_. Each of these models was subsequently used to generate a control population of in silico hiPSC-CMs as in [18,33]. Then we switched on the respective LQT mutation in each of the control population, thus obtaining two mutant populations, expressing LQT1 and LQT2 mutations, respectively. A summary of this approach is reported in Figure S1. Finally, we ran specific analyses on the control and mutant populations to compare them to each other and to the experimental data from the literature.

### 4.2. Control and Mutant Slow Delayed Rectifying Current I_Ks_

We rewrote the I_Ks_ formulation based on the experimental data from [3], where the authors investigated the *KCNQ1* R190Q mutation in hiPSC-CMs. Mutant hiPSC-CMs were produced from father and son, both affected by LQT1, while control cells were produced from two healthy control subjects. In detail, the available data for control and mutant I_Ks_ were: (i) the deactivation time constant, (ii) the steady-state activation curve, (iii) I_Ks_ step current and iv) the I_Ks_ tail current. We modified our original I_Ks_ formulation [20] to fit all this pool of experiments and we simulated the same activation experimental protocol reported in [3]. We kept the same I_Ks_ structure, with only one activation gate, as in Paci et al. [20] and TenTusscher et al. [34]. Such a formulation allowed us to obtain a very good fitting of the current kinetics (Figure 13c,d). However, with only one activation gate, we were not able to perfectly fit both the experimental peak and tail currents (Figure 13a,b). Therefore, we aimed at an acceptable compromise in the fitting of the two features of the current. The resulting mathematical formulations of the control and mutant I_Ks_ are reported in the Supplementary Material, Section 1, while the fitting results are reported in Figure 13.

### 4.3. Control and Mutant Rapid Delayed Rectifying Current I_Kr_

As in the previous section, we also wrote a formulation of the control and mutant current for I_Kr_ based on the experimental data from [4], in which the authors studied the N996I mutation in the *KCNH2* gene. In contrast to I_Ks_ data, in this case, the control hiPSC-CMs were not produced from healthy subjects, but obtained from the mutant cells by targeted gene correction. The available voltage-clamp data are: (i) I_Kr_ step current, (ii) I_Kr_ tail current and (iii) I_Kr_ activation time constant (Figure 14). As in the previous section, our I_Kr_ formulation [20] was adapted to this pool of experimental data to obtain the control and mutant formulations reported in the Supplementary Material, Section 2.

### 4.4. Control Populations (LQT1_CTRL and LQT2_CTRL) of In Silico hiPSC-CMs

We generated two control hiPSC-CM populations: the former using as a baseline model the Paci2018 [20] after control I_Ks_ replacement; the latter after control I_Kr_ replacement. In both cases, we proceeded as in [18,33]. We first generated a random population of in silico hiPSC-CMs by sampling thirteen parameters in the baseline model by means of latin hypercube sampling. The sampled parameters are the maximum conductances/currents of fast and late Na^+^ currents (I_Na_, I_NaL_), funny current (I_f_), L-type Ca^2+^ current (I_CaL_), I_Ks_, I_Kr_, inward rectifying K^+^ current (I_K1_), transient outward K^+^ current (I_to_), Na^+^/Ca^2+^ exchanger (I_NCX_), Na^+^/K^+^ pump (I_NaK_), sarcolemmal Ca^2+^ pump (I_pCa_), SERCA pump (I_SERCA_) and RyR-sensitive Ca^2+^ release from sarcoplasmic reticulum (I_RyR_). Each parameter was sampled in the range [0.5; 2] of its nominal value from [20]. Globally, 10,000 parameter sets (i.e., individual models) were simulated for 800 s to allow the models to reach their steady state; these models represent the random population. The next step consisted of calibrating the random population, i.e., discarding all those models whose AP biomarkers are not in agreement with the experimental data, thus obtaining a subset of models that we called the calibrated population. As in [18], we considered as experimental variability intervals of the AP biomarkers those intervals obtained by merging together six AP biomarkers datasets computed on the spontaneous hiPSC-CM APs: Ma2011 [13], Moretti2010 [3], Ma2013 [6], Fatima2013 [35], Lahti2012 [5] and Kujala2012 [8]. We did not consider the AP biomarkers computed on the control APs in [4] since they were obtained in paced conditions. For each AP biomarker, we considered the union of the ranges mean − 2SD and mean + 2SD from each dataset to get the lower (LB) and upper (UB) bounds of the ranges to which the AP biomarkers computed on the APs of the random population must belong to (Table 5). In case of meaningless bounding values, e.g., negative APD, the respective LB was set to zero. The AP biomarkers considered for the calibration were: (i) rate of spontaneous AP (Rate), (ii) maximum diastolic potential (MDP), (iii) peak potential (Peak), (iv) AP amplitude (APA), (v) maximum upstroke velocity (dV/dt_max_) and (vi) AP duration at different repolarization percentages (APD_xx_). Additionally, models from the random populations were discarded also for the following two reasons: (i) if they did not produce spontaneous APs or (ii) if their cytosolic Na^+^ or sarcoplasmic Ca^2+^ concentrations did not belong to the intervals [5; 15] and [0; 5] mM, respectively. For simplicity, we called the population based on the control I_Ks_ from [3] LQT1_CTRL and the population based on the control I_Kr_ from [4] LQT2_CTRL.

### 4.5. Mutant LQT1 and LQT2 (LQT1_MUT and LQT2_MUT) In Silico Populations

After developing the LQT1_CTRL and the LQT2_CTRL populations, we switched on the respective mutation in each of the hiPSC-CM models, i.e., we replaced the control I_Ks_ and I_Kr_ formulations with their mutant equivalent, obtaining two new mutant populations: LQT1_MUT and LQT2_MUT. In this case, no experimental calibration was performed. However, models were again excluded in cases where (i) the mutation made them stop generating spontaneous APs, or (ii) their cytosolic Na^+^ or sarcoplasmic Ca^2+^ were out of range. Due to the lack of an accepted criterion to separate mutant APs showing pseudo-physiological APD from APs clearly showing the effect of the mutation, as in [18], we divided the LQT1_MUT and the LQT2_MUT populations into two groups using only the AP shapes. Provided that the *KCNQ1* R190Q and the *KCNH2* N996I mutations affect all cells in LQT1_MUT and LQT2_MUT, respectively, we named normal-like the group with APDs more similar to control hiPSC-CMs, and at risk the group with longer APDs. Instead of setting one arbitrary threshold on only one AP biomarker, e.g., APD_90_, we chose to use k-means clustering [36] applied to Rate and to all the APDs. AP biomarkers were standardized with mean = 0 and standard deviation = 1 and k-means was repeated 200 times to stabilize the centroids in the AP biomarker space.

### 4.6. In Silico Drug Tests

Quinidine is a class Ia antiarrhythmic drug, hence blocking I_Na_, but also dramatically affecting other currents, e.g., I_Kr_ and I_CaL_. The effect of three doses of quinidine (1.5, 3, and 9 µM) was tested on all the in silico populations. Drug administration was performed using the single pore block model, as in [18]. The couples (IC_50_ in µM, Hill’s coefficients) for the quinidine effect on I_Na_ and I_CaL_ were taken from [37]: I_Na_ (14.6, 1.22) and I_CaL_ (6.4, 0.68). The couples for I_Kr_, I_Ks_ and I_to_ were taken from [38]: I_Kr_ (0.343, 1), I_Ks_ (4.899, 1.4) and I_to_ (3.487, 1.3). Quinidine was administered from steady state for 250 s to all the models in each population. AP biomarkers were computed on the last 10 APs if arrhythmic events, e.g., EADs, DADs or repolarization failure, did not happen. In this test, we did not exclude any model based on the absence of spontaneous APs or because of out-of-boundaries Na^+^ and Ca^2+^ concentrations, since we aimed to appreciate all the abnormalities induced by quinidine.

## 5. Conclusions

In conclusion, in this work we were able to reproduce the two most common loss-of-function LQT syndromes by means of large-scale simulations, which also enabled the investigation of biophysical mechanisms not easy to assess in vitro, e.g., how the expression of ionic currents can impact on the mutation. Moreover, we provided a tool to assess adverse effects of drugs on the electrophysiology of control and mutant cells, in the perspective of in silico drug tests, not only on adult cells [19], but also on hiPSC-CMs as required in the Comprehensive In vitro Proarrhythmia Assay framework [39].

## Figures and Tables

**Table 1:** Summary of the main AP biomarkers (mean ± SD) for the in silico hiPSC-CM populations. APA: AP amplitude. APD_50_: AP duration at 50% of repolarization. APD_90_: AP duration at 90% of repolarization. dV/dt_max_: maximum upstroke velocity. MDP: maximum diastolic potential. Peak: peak voltage. Rate: rate of the spontaneous APs.

Population (# Models)	Rate (bpm)	MDP (mV)	Peak (mV)	APA (mV)	dV/dt_max_ (V/s)	APD_50_ (ms)	APD_90_ (ms)
LQT1_CTRL (3584)	46 ± 15	−77 ± 3	38 ± 7	114 ± 9	37 ± 21	274 ± 63	344 ± 74
LQT1_MUT (3238)	44 ± 14	−77 ± 3	38 ± 7	114 ± 9	36 ± 21	317 ± 94	389 ± 102
LQT2_CTRL spontaneous (1226)	45 ± 14	−76 ± 3	35 ± 5	111 ± 7	40 ± 21	209 ± 55	254 ± 63
LQT2_MUT spontaneous (1008)	40 ± 12	−77 ± 3	40 ± 4	116 ± 6	41 ± 21	292 ± 58	340 ± 64
LQT2_CTRL paced (979)	60 ± 0	−76 ± 3	37 ± 5	114 ± 7	55 ± 24	233 ± 60	288 ± 72
LQT2_MUT paced (650)	60 ± 0	−76 ± 3	42 ± 4	118 ± 5	52 ± 23	322 ± 62	380 ± 70

**Table 2:** AP biomarkers experimentally recorded in spontaneously beating control and mutant hiPSC-CMs in [3]. AP biomarkers are expressed as mean ± SD. APA: AP amplitude. APD_50_ and APD_90_: AP duration at 50% and 90% of repolarization. dV/dt_max_: maximum upstroke velocity. MDP: maximum diastolic potential. Peak: peak voltage. Rate: rate of the spontaneous APs.

AP Biomarkers	Moretti 2010 [3] Control (*n* = 21)	Moretti 2010 [3] Mutant (*n* = 14)
Rate (bpm)	68 ± 12	60 ± 8
MDP (mV)	−64 ± 10	−65 ± 10
Peak (mV)	44 ± 7	46 ± 8
APA (mV)	108 ± 10	110 ± 10
dV/dt_max_ (V/s)	9 ± 1	8 ± 1
APD_50_ (ms)	323 ± 139	654 ± 328
APD_90_ (ms)	381 ± 162	745 ± 342

**Table 3:** AP biomarkers measured in control and mutant hiPSC-CMs paced at 60 bpm by Bellin et al. [4]. AP biomarkers are expressed as mean ± SD. APA: AP amplitude. APD_50_ and APD_90_: AP duration at 50% and 90% of repolarization, respectively. dV/dt_max_: maximum upstroke velocity. MDP: maximum diastolic potential. Peak: peak voltage. Rate: stimulation frequency.

AP biomarkers	Bellin 2013 [4] Control (*n* = 10)	Bellin 2013 [4] Mutant (*n* = 14)
Rate (bpm)	60 ± 0	60 ± 0
MDP (mV)	−75 ± 6	−75 ± 6
APA (mV)	116 ± 10	120 ± 9
dV/dt_max_ (V/s)	71 ± 39	66 ± 42
APD_50_ (ms)	164 ± 78	227 ± 66
APD_90_ (ms)	207 ± 92	292 ± 81

**Table 4:** Percent of models in each population showing abnormalities in response to increasing doses of quinidine. The values in brackets in the LQT1_MUT and LQT2_MUT columns represent the percent of models producing abnormalities in the at risk and normal-like groups in the respective mutant population.

Dose (µM)	LQT1_CTRL	LQT1_MUT (at Risk vs. Normal-Like)	LQT2_CTRL	LQT2_MUT (at Risk vs. Normal-Like)
0	0%	0% (0% vs. 0%)	0%	0% (0% vs. 0%)
1.5	5%	11% (16% vs. 7%)	1%	1% (2% vs. 0%)
3	11%	23% (29% vs. 18%)	4%	6% (10% vs. 4%)
9	35%	54% (55% vs. 53%)	46%	46% (50% vs. 44%)

**Table 5:** Datasets used for calibrating the random populations. Lower (LB) and upper (UB) bounds for each AP biomarker are reported in columns two and three. Missing data are marked as ---. This table has been reproduced from the Supplementary Material of [18].

Dataset (# Cells)			Ma2011 [13] (32)		Moretti2010 [3] (21)		Ma2013 [6] (12)		Fatima2013 [35] (6)		Lahti2012 [5] (13)		Kujala2012 [8] (16)	
AP biomarkers	LB	UB	Mean	SD	Mean	SD	Mean	SD	Mean	SD	Mean	SD	Mean	SD
Rate (bpm)	>0	209	35	12	68	12	69	39	118	45	72	22	41	24
MDP (mV)	−89	−44	−76	7	−64	10	−61	5	−64	6	−63	5	−68	7
Peak (mV)	17	58	28	6	44	7	---	---	39	3	---	---	---	---
APA (mV)	76	139	104	6	108	10	86	5	102	5	113	9	118	10
dV/dt_max_ (V/s)	>0	82	28	27	9	1	13	16	24	12	27	23	---	---
APD_10_ (ms)	20	128	74	27	---	---	---	---	---	---	---	---	---	---
APD_20_ (ms)	>0	290	---	---	---	---	138	76	---	---	---	---	---	---
APD_30_ (ms)	59	301	180	61	---	---	---	---	---	---	---	---	---	---
APD_50_ (ms)	>0	601	---	---	323	139	338	115	175	106	265	54	204	81
APD_70_ (ms)	146	631	---	---	---	---	388	121	---	---	---	---	---	---
APD_90_ (ms)	1	705	415	123	381	162	434	108	298	148	314	63	330	90

## Supplementary Materials

Supplementary materials can be found at http://www.mdpi.com/1422-0067/19/11/3583/s1, Figure S1: Flowchart for generating the four in silico populations LQT1_CTRL, LQT1_MUT, LQT2_CTRL, LQT2_MUT, Figure S2: Magnification of the action potentials included in the two populations built using the IKs experiments by Moretti et al. [2]: (a) LQT1_CTRL; (b) LQT1_MUT, Figure S3: AP biomarker distributions in the LQT1_CTRL (cyan) and LQT1_MUT (magenta) populations, Figure S4: Magnification of the action potentials included in the two populations built using the IKr experiments by Bellin et al. [3]: (a) LQT2_CTRL; (b) LQT2_MUT, Figure S5: AP biomarker distributions in the non-paced (cyan) and paced (blue) LQT2_CTRL and non-paced (magenta) and paced (red) LQT2_MUT populations, Figure S6: Parameter distribution in the 21 LQT1_MUT models showing an APD90 prolongation greater than 50% as consequence of IKs loss-of-function, Figure S7: Original IKs peak current from the Paci2018 model (dashed trace), Table S1: Residual percent currents after Quinidine administration.

## Abbreviations

AP	Action potential
APD_xx_	Action potential duration at xx%
hiPSC	Human induced pluripotent stem cell
APA	Action potential amplitude
DAD	Delayed afterdepolarization
dV/dt_max_	Maximum upstroke velocity
EAD	Early afterdepolarization
EHT	Engineered heart tissue
FDA	Food and drug administration
hiPSC-CM	Human induced pluripotent stem cell-derived cardiomyocyte
I_CaL_	L-type Ca^2+^ current
I_f_	Funny current
I_Kr_	Rapid delayed rectifying K^+^ current
I_Ks_	Slow delayed rectifying K^+^ current
I_K1_	Inward rectifying K^+^ current
I_Na_	Fast Na^+^ current
I_NaK_	Na^+^/K^+^ pump
I_NaL_	Late Na^+^ current
I_NCX_	Na^+^/Ca^2+^ exchanger
I_pCa_	Sarcolemmal Ca^2+^ pump
I_RyR_	RyR-sensitive Ca^2+^ release
I_SERCA_	SERCA pump
I_to_	Transient outward K^+^ current
LB	Lower bound
LQT1_CTRL	hiPSC-CM population with control I_Ks_ from [3]
LQT1_MUT	hiPSC-CM population with mutant I_Ks_ from [3]
LQT2_CTRL	hiPSC-CM population with control I_Kr_ from [4]
LQT2_MUT	hiPSC-CM population with mutant I_Kr_ from [4]
MDP	Maximum diastolic potential
mean	Mean value
Paci2018	hiPSC-CM model from [20]
Peak	Peak potential
Rate	Spontaneous action potential rate
SD	Standard deviation
UB	Upper bound
ΔAPD_90_%	Percent APD_90_ variation
