# Supplementary material for: Large-Scale Simulation of the Phenotypical Variability Induced by Loss-of-Function Long QT Mutations in Human Induced Pluripotent Stem Cell Cardiomyocytes
Source: Int J Mol Sci. 2018 Nov 13;19(11):3583. doi: 10.3390/ijms19113583 (PMC6274824; doi:10.3390/ijms19113583)
Supplement: Supplementary file 1 [file ijms-19-03583-s001.zip › 20181113_Supplementary Material.docx]

Supplementary Material

Large-scale simulation of the phenotypical variability induced by loss-of-function Long QT mutations in human induced pluripotent stem cell cardiomyocytes

Michelangelo Paci, Simona Casini, Milena Bellin, Jari Hyttinen and Stefano Severi

*V*: membrane potential in *V*. *VmV*: membrane potential in *mV*. *time*: simulation time in *s*.

All the time constants in *ms*. All the ionic concentrations in *mM*. All the reversal potentials in *V*.

For the constant values, other state variable and ionic current formulations here not reported, refer to [1].

1. Formulation of the control and mutant I_Ks_

$$I_{Ks}=g_{Ks}\cdot Xs^{2}\cdot\left(1+\frac{0.6}{1+\left(\frac{3.8e-5}{Ca_{i}}\right)^{1.4}}\right)\cdot\left(V-E_{Ks}\right)$$

$$E_{Ks}=\frac{R\cdot T}{F}\cdot\ln\frac{K_{o}+P_{kna}\cdot Na_{o}}{K_{i}+P_{kna}\cdot Na_{i}}$$

$$\frac{\text{d}Xs}{\text{d}time}=1000\cdot\frac{Xs_{inf}-Xs}{\tau_{Xs}}$$

*1.1. Control I_Ks_*

$$g_{Ks}=\text{19.2168 }(S/F)$$

$$Xs_{inf}=\frac{1}{1+e^{\frac{-13.71-VmV}{10.38}}}$$

$$\alpha_{Xs}=0.95\cdot\frac{1100}{\sqrt{1+e^{\frac{-24-VmV}{6}}}}$$

$$\beta_{Xs}=\frac{1}{1+e^{\frac{VmV-60}{20}}}$$

$$\tau_{Xs}=\alpha_{Xs}\cdot\beta_{Xs}$$

*1.2. Mutant I_Ks_*

$$g_{Ks}=0.25\cdot\text{19.2168 }(S/F)$$

$$Xs_{inf}=\frac{1}{1+e^{\frac{-10.769-VmV}{12.86}}}$$

$$\alpha_{Xs}=\frac{3226}{\sqrt{1+e^{\frac{-11.9-VmV}{6.581}}}}$$

$$\beta_{Xs}=\frac{1}{1+e^{\frac{VmV-58.13}{24.08}}}$$

$$\tau_{Xs}=\alpha_{Xs}\cdot\beta_{Xs}$$

*1.3. Intracellular and extracellular concentrations for voltage-clamp experiments only*

$$Na_{i}=4 (mM)$$

$$K_{i}=145 (mM)$$

$$Ca_{i}=0.0002 (mM)$$

$$Na_{o}=120 (mM)$$

$$K_{o}=4.2 (mM)$$

2. Formulation of the control and mutant I_Kr_

$$I_{Kr}=g_{Kr}\cdot\sqrt{\frac{K_{o}}{5.4}}\cdot Xr1\cdot Xr2\cdot\left(V-E_{K}\right)$$

$$E_{K}=\frac{R\cdot T}{F}\cdot\ln\frac{K_{o}}{K_{i}}$$

$$V_{half,Xr1}=1000\cdot\left(-\frac{R\cdot T}{F\cdot Q}\cdot ln\left(\frac{\left(1+\frac{Ca_{o}}{2.6}\right)^{4}}{L_{0}\cdot\left(1+\frac{Ca_{o}}{0.58}\right)^{4}}\right)-0.033\right)$$

$$\frac{\text{d}Xr1}{\text{d}time}=1000\cdot\frac{Xr1_{inf}-Xr1}{\tau_{Xr1}}$$

$$\frac{\text{d}Xr2}{\text{d}time}=1000\cdot\frac{Xr2_{inf}-Xr2}{\tau_{Xr2}}$$

*2.1. Control I_Kr_*

$$g_{Kr}=380 (S/F)$$

$$Xr1_{inf}=\frac{1}{1+e^{\frac{V_{half,Xr1}-VmV}{8.3}}}$$

$$\alpha_{Xr1}=\frac{36.46}{1+e^{\frac{-37.24-VmV}{2.535}}}$$

$$\beta_{Xr1}=\frac{26.29}{1+e^{\frac{VmV+28.4}{7.887}}}$$

$$\tau_{Xr1}=\alpha_{Xr1}\cdot\beta_{Xr1}$$

$$Xr2_{inf}=\frac{1}{1+e^{\frac{VmV+89}{30}}}$$

$$\alpha_{Xr2}=\frac{3}{1+e^{\frac{-60-VmV}{20}}}$$

$$\beta_{Xr2}=\frac{1.12}{1+e^{\frac{VmV-60}{20}}}$$

$$\tau_{Xr2}=\left\{\begin{matrix} 5\cdot\alpha_{Xr2}\cdot\beta_{Xr2}, if \left(Xr2_{inf}\geq Xr2\right) \\ \alpha_{Xr2}\cdot\beta_{Xr2}, \text{o}therwise \end{matrix}\right.$$

*2.1. Mutant I_Kr_*

$$g_{Kr}=280 (S/F)$$

$$Xr1_{inf}=\frac{1}{1+e^{\frac{V_{half,Xr1}-VmV}{10.3}}}$$

$$\alpha_{Xr1}=\frac{196.3}{1+e^{\frac{-36.84-VmV}{2.597}}}$$

$$\beta_{Xr1}=\frac{6.806}{1+e^{\frac{VmV+37.59}{10.9}}}$$

$$\tau_{Xr1}=\alpha_{Xr1}\cdot\beta_{Xr1}$$

$$Xr2_{inf}=\frac{1}{1+e^{\frac{VmV+89}{29}}}$$

$$\alpha_{Xr2}=\frac{3}{1+e^{\frac{-60-VmV}{20}}}$$

$$\beta_{Xr2}=\frac{1.12}{1+e^{\frac{VmV-60}{20}}}$$

$$\tau_{Xr2}=\left\{\begin{matrix} 6\cdot\alpha_{Xr2}\cdot\beta_{Xr2}, if \left(Xr2_{inf}\geq Xr2\right) \\ \alpha_{Xr2}\cdot\beta_{Xr2}, \text{o}therwise \end{matrix}\right.$$

*2.3. Intracellular and extracellular concentrations for voltage-clamp experiments only*

$$K_{i}=155 (mM)$$

$$K_{o}=5.4 (mM)$$

3. Supplementary figures and tables


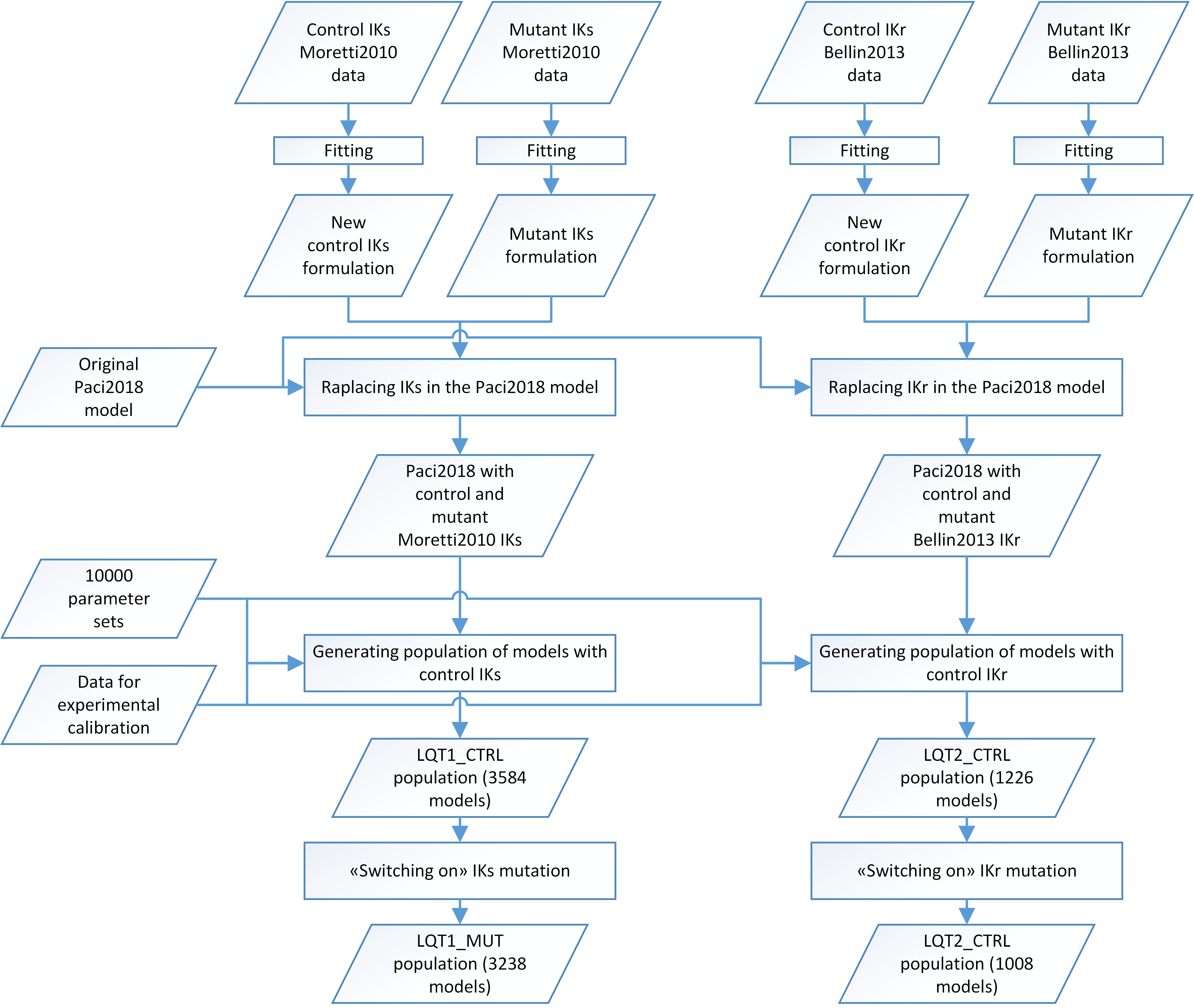


**Figure S1.** Flowchart for generating the four *in silico* populations LQT1_CTRL, LQT1_MUT, LQT2_CTRL, LQT2_MUT. Moretti2010 patch clamp experiments from [2]. Bellin2013 patch clamp experiments from [3].


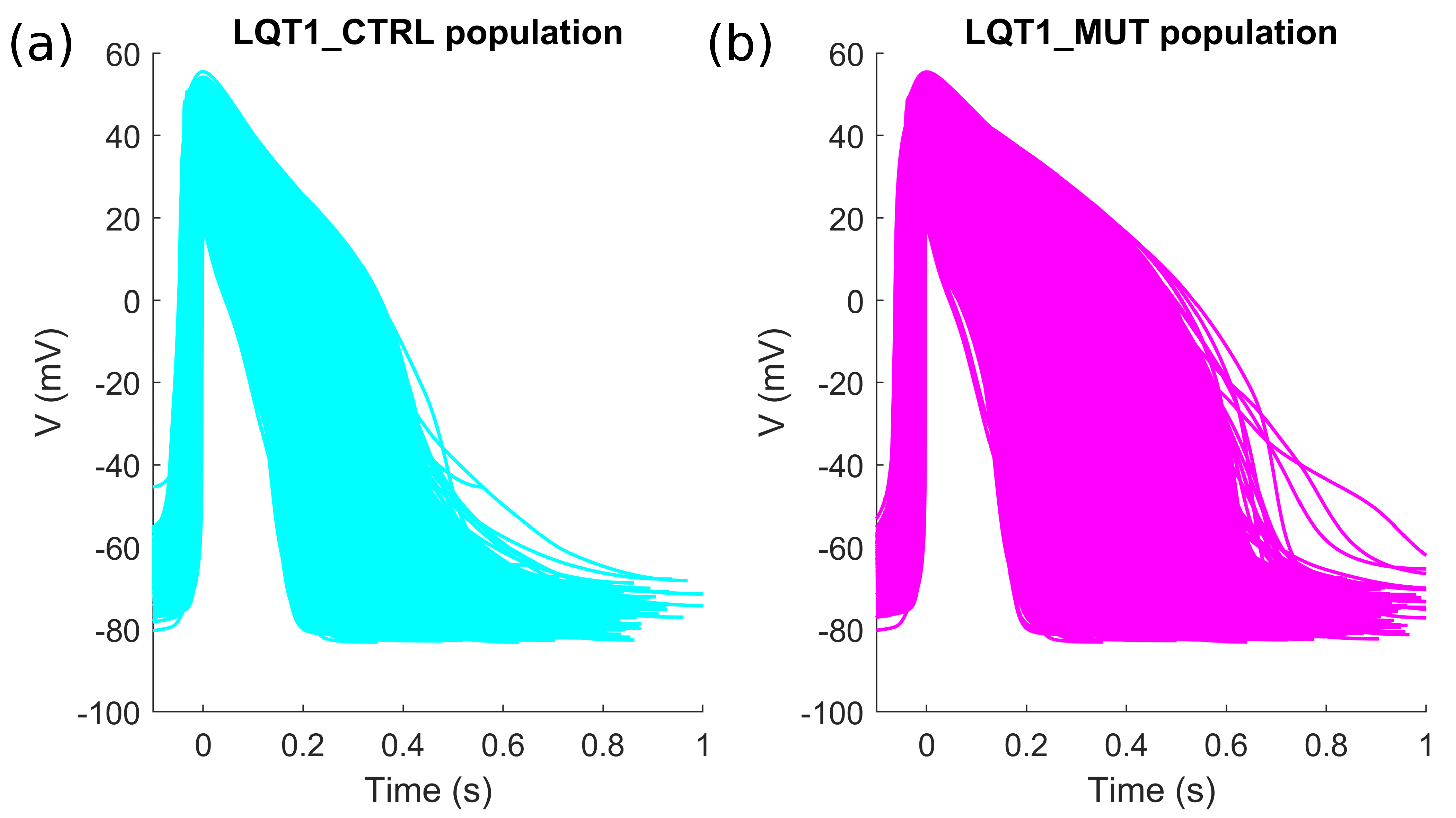


**Figure S2.** Magnification of the action potentials included in the two populations built using the I_Ks_ experiments by Moretti *et al.*[2]: (**a**) LQT1_CTRL; (**b**) LQT1_MUT.


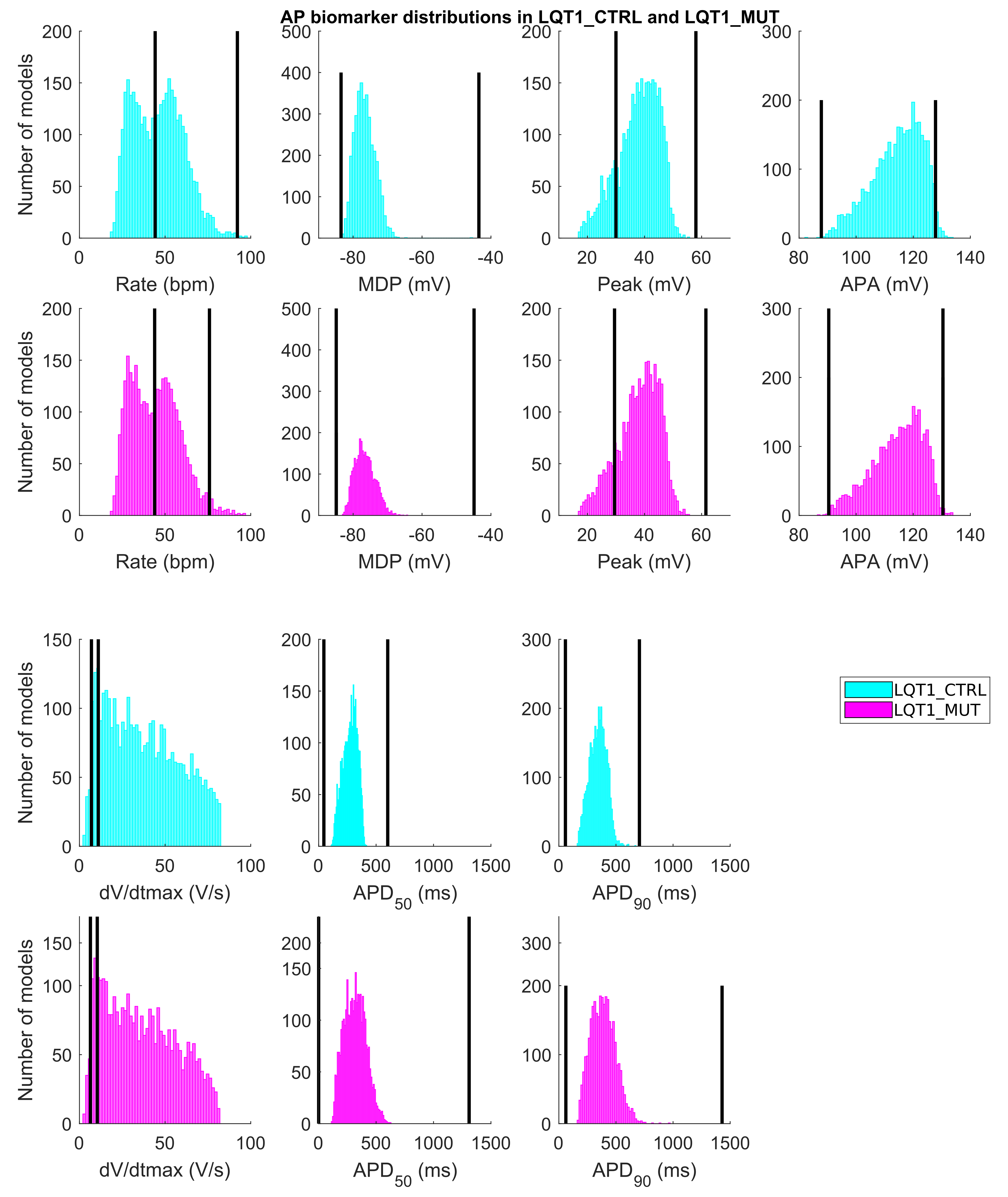


**Figure S3.** AP biomarker distributions in the LQT1_CTRL (cyan) and LQT1_MUT (magenta) populations. The black lines represents the lower bound (mean-2SD) and upper bound (mean+2SD) for each AP biomarker from the Moretti2010 [2] control dataset (see Table 2 in the main paper).


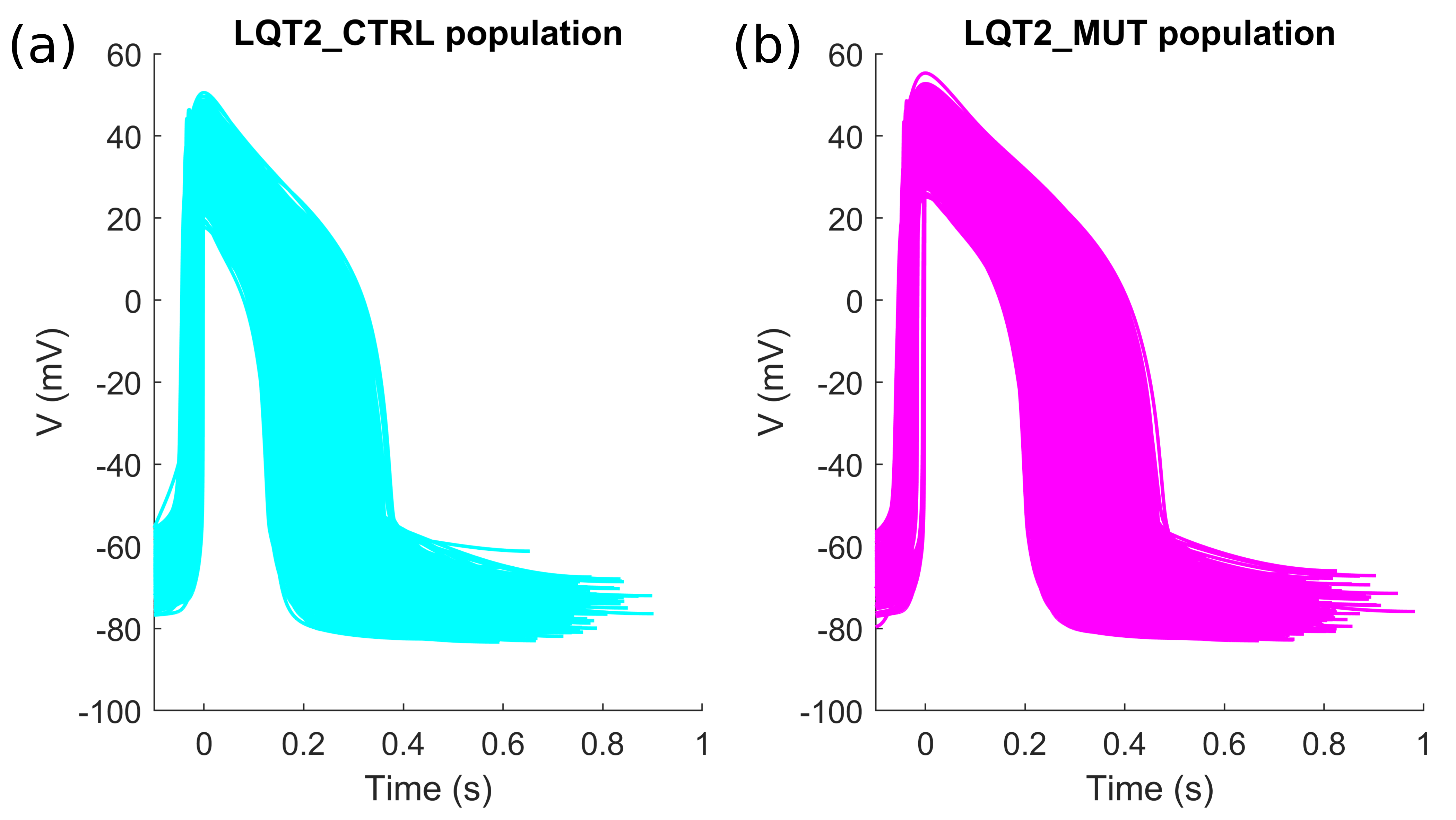


**Figure S4.** Magnification of the action potentials included in the two populations built using the I_Kr_ experiments by Bellin *et al.* [3]: (a) LQT2_CTRL; (b) LQT2_MUT.


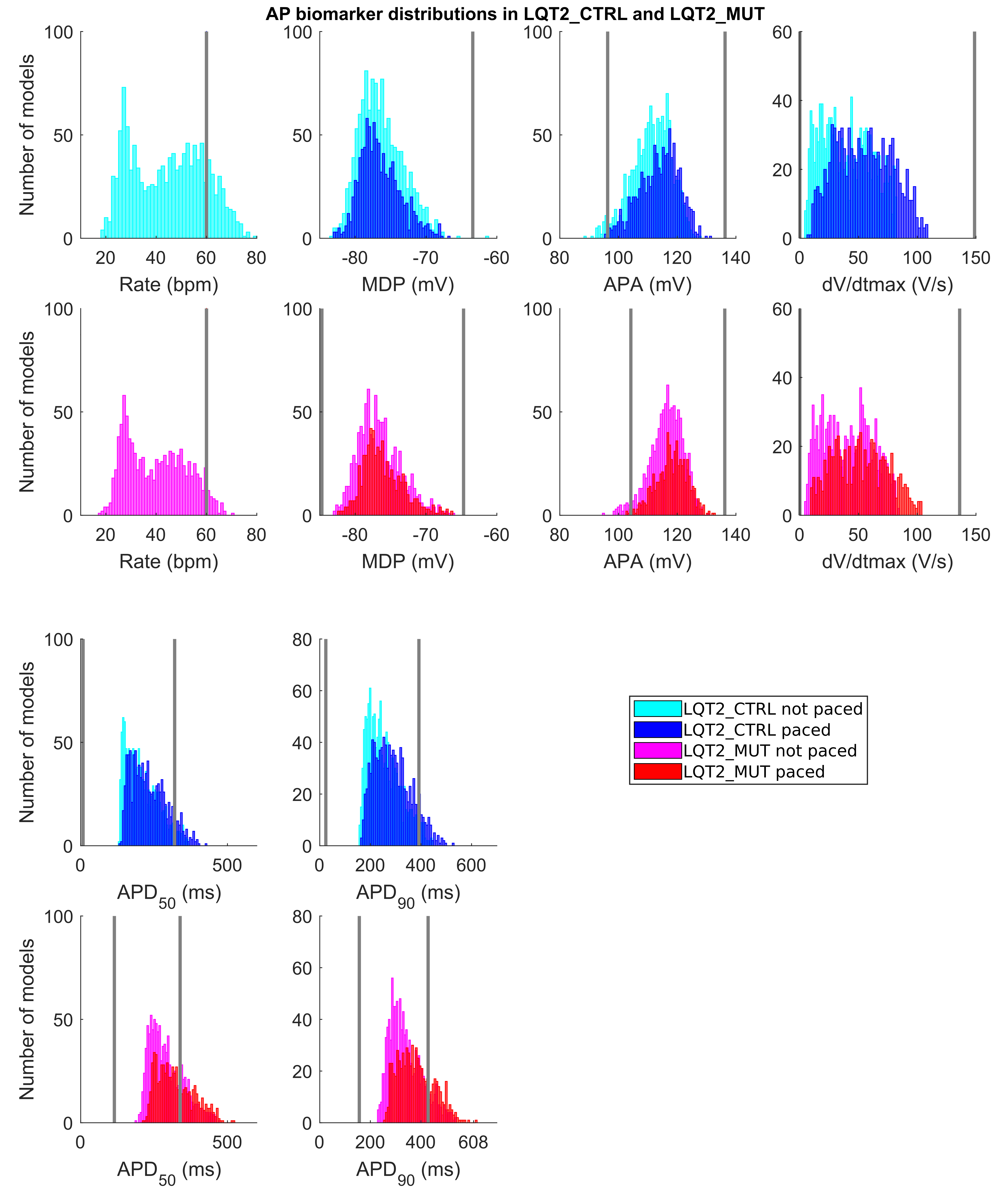


**Figure S5.** AP biomarker distributions in the non-paced (cyan) and paced (blue) LQT2_CTRL and non-paced (magenta) and paced (red) LQT2_MUT populations. The gray lines represents the lower bound (mean-2SD) and upper bound (mean+2SD) for each AP biomarker from the Bellin2013 control and mutant datasets [3], reported in Table 3 in the main paper. For the Rate AP biomarker we reported only the distribution in case of spontaneous APs, since the external pacing rate was set to 60 bpm for all the models.


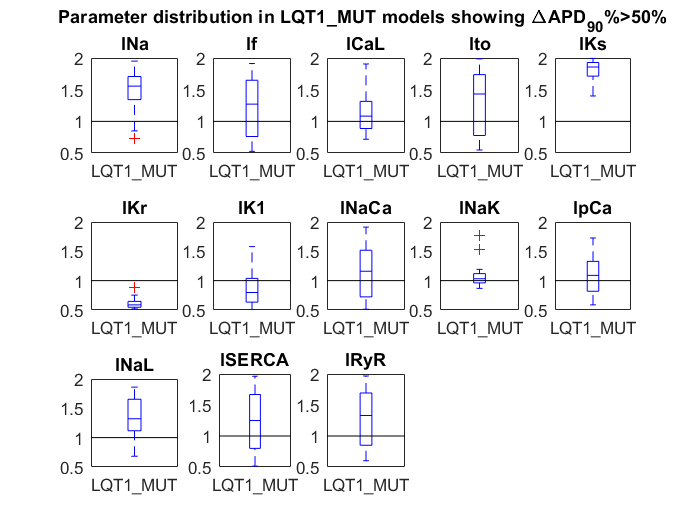


**Figure S6.** Parameter distribution in the 21 LQT1_MUT models showing an APD_90_ prolongation greater than 50% as consequence of I_Ks_ loss-of-function.


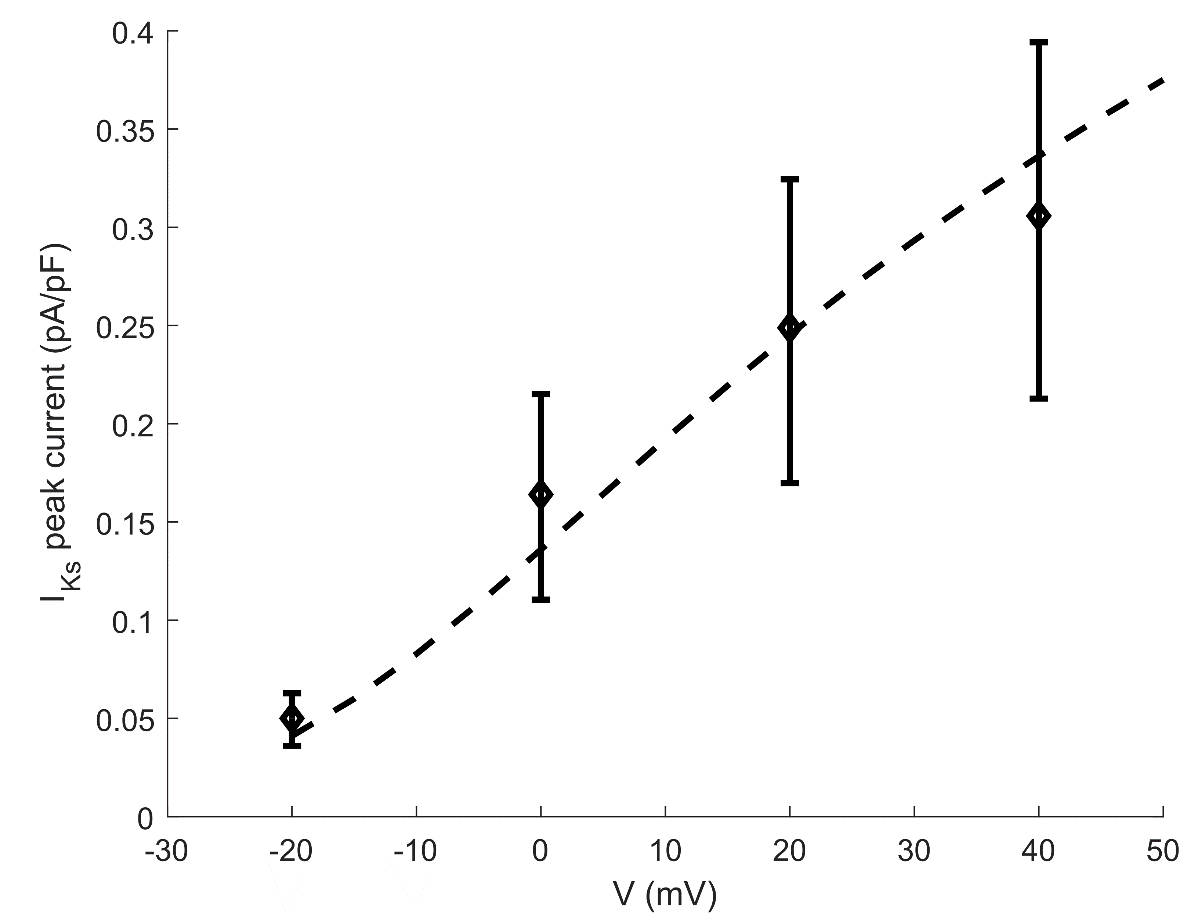


**Figure S7.** Original I_Ks_ peak current from the Paci2018 model (dashed trace). Black diamonds represent the experimental data from Ma *et al.* [4].

**Table S1.** Residual percent currents after Quinidine administration

| **Dose (µM)** | **I_Na_** | **I_CaL_** | **I_Kr_** | **I_Ks_** | **I_to_** |
| --- | --- | --- | --- | --- | --- |
| 1.5 | 94% | 73% | 19% | 84% | 75% |
| 3 | 87% | 63% | 11% | 67% | 55% |
| 9 | 64% | 44% | 4% | 30% | 23% |

References

1. Paci M et al. Automatic optimization of an in silico model of human iPSC derived cardiomyocytes recapitulating calcium handling abnormalities. Front Physiol. 2018;9(June):709.

2. Moretti A et al. Patient-specific induced pluripotent stem-cell models for long-QT syndrome. N Engl J Med. 2010;363(15):1397–409.

3. Bellin M et al. Isogenic human pluripotent stem cell pairs reveal the role of a KCNH2 mutation in long-QT syndrome. EMBO J. 2013;32(24):3161–75.

4. Ma J et al. High purity human-induced pluripotent stem cell-derived cardiomyocytes: electrophysiological properties of action potentials and ionic currents. AJP - Hear Circ Physiol. 2011;301(5):H2006–17.
